# Administration of Glucose at Litter Equalization as a Strategy to Increase Energy in Intrauterine Growth Restricted Piglets

**DOI:** 10.3390/ani10071221

**Published:** 2020-07-17

**Authors:** Joanna Klaaborg, Charlotte Amdi

**Affiliations:** 1 Department of Veterinary and Animal Sciences, Faculty of Health and Medical Sciences, University of Copenhagen, 1870 Frederiksberg C, Denmark; 2 Danish Meat Research Institute, Danish Technological Institute, Gregersensvej 9, 2630 Taastrup, Denmark; joannaklaaborg@yahoo.dk

**Keywords:** Glucose administration, hyper-prolific sows, intrauterine growth restriction, piglet survival, rectal temperature

## Abstract

**Simple Summary:**

Hyper-prolific sows with large litters require extra management in order to reduce piglet mortality. One of the reasons is high piglet birth weight variability in these large litters where piglets can range from 300 g to 2.5 kg in the same litter. In this study the strategy of giving energy at litter equalization to the smallest piglets was investigated as this is when most farmers handle the piglets for the first time. The treatments consisted of a control, oral and injected supplementation. There were no differences between the treatments of the piglets suggesting that it is too late to intervene at litter equalization, and if extra management actions are to have an effect then they most likely have to be given already at birth. More research is needed on how to handle the small and underdeveloped piglets in order to reduce piglet mortality.

**Abstract:**

Hyper-prolific sows give birth to large litters and up to 25% of piglets born have been subjected to intrauterine growth restriction (IUGR). The aim of this study was to test whether an oral administration of glucose impacts the survival rate and body weight gain of IUGR piglets at weaning. Different methods (injection versus oral administration of glucose 6 mL or 12 mL, respectively) were tested on IUGR piglets at litter equalization (i.e., when piglets are handled the first time at 5–20 h after birth). Injecting glucose generated the highest whole-blood glucose level + 3 h after treatment, however, after this no differences were observed. Of the 237 IUGR piglets studied, 98 piglets died or were removed from the nurse sow (41%). Rectal temperature at litter equalization (0 h) was related to the survival of the piglets with an average temperature of 37.1 ± 0.1 °C in surviving piglets and 36.6 ± 0.1 °C in piglets that died. In conclusion, providing these extra management actions at litter equalization is too late to help piglets that have a low rectal temperature and are low on energy. More research investigating different management methods to deal with IUGR piglets are needed as many of these underdeveloped piglets will not survive.

## 1. Introduction

Selection for hyper-prolific sows has caused litter size to increase, however, this has also resulted in lower piglet birth weights and an increase in the number of small piglets. In addition, there is an increasing within-litter piglet birth weight variation with larger litters and a high within-litter piglet birth variation is negatively correlated with survival up to weaning [1]. Some if not all piglets below 1 kg have suffered from intrauterine growth restriction (IUGR) as can be seen on the brain to body ratio [2] due to uterine crowding [3]. IUGR piglets have suffered from impaired growth and development of their organs during gestation and can easily be identified by their unique head-shape [2,4], due to the brain-sparing effect [5]. Accordingly, approximately 25% of piglets in Denmark are IUGR piglets [2,6]. IUGR piglets have a higher mortality as they are smaller and weaker at birth [2,6]. Furthermore, they are challenged during lactation, because they are one week behind in growth compared to their normal littermates [7]. For that reason, they are both a welfare and an economical concern. Although nutritional strategies to reduce piglet variation during gestation are being investigated, the problem of a high mortality in IUGR piglets will not disappear anytime soon. However, interventions at birth might increase their survival rate.

At birth, IUGR piglets have a lower rectal temperature by up to 1 °C [8] and lower glycogen deposits in their liver [2] compared to their normal littermates. This compromises their ability to reach the udder and compete to get the colostrum, which is vital for their growth and survival. Several studies have been conducted to improve IUGR piglets’ survival and growth including performing split nursing [9], providing additional heat and colostrum after birth and placing them with a nurse sow suitable for rearing small piglets [10,11]. Furthermore, an oral supplementation of two commercial products serving as an energy booster has been shown to increase survival rate during lactation [12,13]. For the same reason, some herds choose to give piglets glucose instead of commercial products, as this is less expensive. An energy boost at birth by injecting glucose has also been shown to increase IUGR piglets’ weaning weight by 800 g compared to a control IUGR group [14]. This is most likely because IUGR piglets injected with glucose had more energy to compete at the udder or to put towards growth [14]. Glucose administration could be a practice on farm to increase IUGR piglets’ growth potential and survival rate. However, injections are invasive and they create a point of entry for bacteria [15]. If glucose was to be administered using a less invasive method, it could be a tool to improve the survival and growth performance of IUGR piglets without compromising welfare or health.

Therefore, the aim of this study was to investigate the effect of giving IUGR piglets oral glucose at litter equalization (5–20 h after birth) compared to a glucose injection or water bolus on survival rate and body weight (BW) gain during lactation in a commercial herd. We hypothesized that giving glucose to IUGR piglets at litter equalization would increase survival rate and BW gain during lactation and that oral glucose would be equally effective as glucose injections.

## 2. Materials and Methods

### 2.1. Ethical Approval

The experiment was carried out with approval from the Danish Experimentation Inspectorate, j.nr. 2018-15-0201-01515.

### 2.2. Animals and Experimental Design

The experiment was conducted on a commercial Danish herd with 880 Danish Landrace × Danish Yorkshire sows (DanBred, 2730-Herlev, Denmark) from December 2018 to July 2019 and it included 237 piglets from 104 parity 1–7 sows. Piglets were studied from litter equalization (i.e., when piglets are handled the first time at 5–20 h after birth) to the age of 28 days (i.e., before weaning at 31 days). Piglets were identified as IUGR according to BW and head-shape based on a modified version of Hales et al. [6] and Chevaux et al. [4]. The piglets were defined by visual score as IUGR if they had a steep dolphin-like forehead and additionally either (1) bulging eyes, (2) hair with no direction of growth or (3) an asymmetric body with narrow hindquarters compared to the front shoulders. In addition, it was a requirement that all piglets in the study had a BW between 500–1000 g.

Each week the selection of piglets occurred at arrival on the farm each Tuesday morning before litter equalization. At arrival, all piglets that had been born during the previous 24 h were enclosed in the creep area with a heating lamp (100 Watt) along with their birth littermates for a minimum of one hour. Out of these piglets, 16 IUGR piglets were selected, earmarked and immediately placed together at a nurse sow suitable for rearing small piglets. Nineteen nurse sows were included in the study over a 22-week period. Some weeks only 8 or 12 IUGR piglets could be found and the nurse sow was supplemented with non-experimental piglets. Requirements of the nurse sow included that they had farrowed during the previous 24 h, not been subjected to medical treatment related to farrowing, they were parity 1–2 and had a minimum of 14 teats placed in a small range. When all 16 IUGR piglets were placed together at the same nurse sow, each piglet was weighed using a digital weight (Bjerringbro Vægte ApS, 8850-Bjerringbro, Denmark), rectal temperature was taken with a digital thermometer (Apotekets Digital-termometer, Apotekernes A.m.b.a., 2740-Skovlunde, Denmark) and blood glucose level was measured using a glucose monitor and strips (Accu-Chek Aviva, Roche, 68305-Mannheim, Germany). A drop of blood was obtained by gently puncturing the ear vein with a 23G needle (Kruuse, 5550-Langeskov, Denmark). Afterwards, piglets were appointed to the specific treatment, so the experiment was balanced according to BW, and the treatment was administered at 0 h and + 3 h. Rectal temperature and blood glucose were measured before each treatment. Body weight was also measured before each treatment, and also on days 7, 14, 21 and 28 of lactation. The parity and number of liveborn/stillborn piglets were registered of the birth sow of the piglets. Additionally, parity and number of liveborn/stillborn of the nurse sow as well as sex of the piglet were registered.

The piglets were assigned to one of four treatments (*n* = 60): 6 mL warmed (37 °C) water given orally with a 12 mL syringe (WATER), 1.5 mL glucose (B. Braun, 34209-Melungen, Germany) (50 mg/mL) injected subcutaneously in four places (two regions); two in the neck and two in the groin (INJECT), 6 mL glucose (50 mg/mL) given orally with a 12 mL syringe (GLUC6) or 12 mL glucose (50 mg/mL) given orally with a 12 mL syringe (GLUC12). Oral glucose was administered by putting the syringe in the piglets’ mouth and having them drink it slowly until the syringe was empty. This method resulted in some spilling. However, it was chosen as it was not invasive and a method than can easily be practiced by farm employees. After each piglet had been subjected to treatment, it was placed in the enclosed creep area and when all piglets in the litter had received treatment, the entry to the creep area was opened and piglets could access the sow.

### 2.3. Housing and Management

During the experiment, all animals were managed in accordance with the standard routines of the herd. Every day, the stock personnel inspected health of the animals and usual practices for treatments, vaccinations and management were followed. Sows were artificially inseminated with mixed DanBred duroc semen (Hatting Agro, 8700-Horsens, Denmark). One week before expected farrowing, sows were moved to the farrowing unit. The farrowing unit consisted of 6 sections with each 36 individual pens. The experiment included 13 batches. Accordingly, all sections were included in the experiment. Farrowing pens were Free-Farrowing pens (Jyden Bur A/S, 7570-Vemb, Denmark), which meant that sows were loose from insertion in the farrowing stable until weaning. Pens measured 3.0 m in length and 2.0 m in width equaling to 6.0 m^2^ and had 67% solid concrete floor and 33% slatted cast iron floor. The creep area was 0.86 m^2^. Each section was ventilated using negative pressure and inlet from diffuse ceiling. One ceiling outlet was installed in each section with pit ventilation and temperature was adjusted to 20 °C in week 1, 19 °C in week 2 and 18 °C in week 3 and 4. There was floor heating in the creep area and in the aisle, which was set to 41 °C. Additionally, there was a heating lamp above the creep area, which was turned on from two days before expected farrowing to when piglets were observed to be mostly outside the creep area after 7 days approximately.

Sows observed with illness during lactation were treated. On the day of birth, piglets were subjected to iron injections (200 mg/mL, Viloferron^®^, 2840-Holte, Denmark) and tooth grinding and their umbilical cords were clipped and disinfected (1 mL/100 kg, Cyclo Spray Vet, Dechra, 7171-Uldum, Denmark). When male piglets were 3–5 days old, they were surgically castrated using local anesthetic (5 mg/mL, Procamidor Vet, Salfarm Danmark A/S, 6000-Kolding, Denmark) beforehand and given pain relief (20 mg/mL, Melovem, Salfarm Danmark A/S, 6000-Kolding, Denmark) afterwards. If a piglet died or was removed from the nurse sow during the experiment, the ear tag was removed and the date and reason (i.e., crushed, hunger/weak, legs, diarrhoea or other) was registered. Piglets were weaned at 31 days of age.

### 2.4. Feeding

Sows were fed a lactation diet consisting of 39.3% wheat, 39.3% barley and 14.8% soy bean meal (13.5 Metabolizable Energy (ME)/kg, crude protein 15.5%, crude fat 4.3%, fibre 3.5% per kg). Level of lysine/kg was 8.6 g/kg feed. Sows were fed three times per day at 07:30 h, 13:00 h and 20:00 h and were given 3.3 kg/day on the day of farrowing increasing to a maximum of 13.3 kg/day on day 24 in lactation and for the remaining lactation period. Feed level was adjusted individually every second day according to whether leftovers were present in the trough. Furthermore, sows had free access to water and straw from a rack. Piglets were creep fed from 10 days of age on the floor. They were given approximately a handful per day.

### 2.5. Blood Samples

Blood samples were taken on day 7 in lactation by holding the piglets in dorsal recumbency and drawing 6 mL of blood from the jugular vein using 22G needles (BD Vacutainer, Belliver Industrial Estate, 53073-Plymouth, United Kingdom) into Vacutainer tubes containing EDTA (BD Vacutainer, Belliver Industrial Estate, 53073-Plymouth, United Kingdom). Samples were placed on ice until centrifugation, which occurred in room temperature for 15 min at 1500× *g* (CM-6MT, Elmi, 49276-Riga, Latvia). Afterwards, the plasma was transferred to Eppendorf tubes (Sarstedt, 51588-Nümbrecht, Germany) and placed on ice until approximately 1 h later when it was frozen at −20 °C for later analysis of plasma insulin-like growth factor 1 (IGF-1). Plasma IGF-1 concentrations were measured using a porcine IGF-1 ELISA kit (Nordic Biosite, Tæby, Sweden) according to manufacturer’s recommendations and performed in duplicates.

### 2.6. Statistical Analysis

The effect of treatment and time (hour and day) on blood glucose, body weight and temperature were studied using linear mixed models in R with the piglet as the experimental unit. The following model was used to examine piglet body weight (BW):

(1) $$Y_{ijklmn}=\mu +\alpha_{i}+\beta_{j}+{\left(\alpha \beta \right)}_{ij}+\gamma x_{k}+A_{k}+B_{l}+C_{m}+D_{n}+\delta_{jk}+e_{ijklmn}$$

where $Y_{ijklmn}$ is the response variable, μ is the intercept, $\alpha_{i}$ is the fixed effect of treatment (i = WATER, INJECT, GLUC6, GLUC12), $\beta_{j}$ is the fixed effect of time (j = 1, 7, 14, 21, 28), $\gamma x_{k}$ is piglet birth weight as a covariate, $A_{k}$ is the random effect of piglet, ${Β}_{l}$ is the random effect of birth sow, $C_{m}$ is the random effect of nurse sow, $D_{n}$ is the random effect of batch, $\delta_{jk}$ is a random autocorrelated term accounting for repeated measurements and $e_{ijklmn}$ is the residual error.

The following model was used to examine temperature and blood glucose:

(2) $$Y_{ijklmn}=\mu +\alpha_{i}+\beta_{j}+{\left(\alpha \beta \right)}_{ij}+A_{k}+B_{l}+C_{m}+D_{n}+\delta_{jk}+e_{ijklmn}$$

where $Y_{ijklmn}$ is the response variable, μ is the intercept, $\alpha_{i}$ is the fixed effect of treatment (i = WATER, INJECT, GLUC6, GLUC12), $\beta_{j}$ is the fixed effect of time (j = 0, 3), $A_{k}$ is the random effect of piglet, ${Β}_{l}$ is the random effect of birth sow, $C_{m}$ is the random effect of nurse sow, $D_{n}$ is the random effect of batch, $\delta_{jk}$ is a random autocorrelated term accounting for repeated measurements and $e_{ijklmn}$ is the residual error. In order to ensure homogeneity of variance, BW and blood glucose were transformed by logarithm before analysis and then back-transformed. For the effect of temperature and glucose on survival and the effect of treatment on IGF-1 levels the statistical software SAS was used (proc mixed; SAS Inst.Inc., Cary, NC, USA) with the piglet as the experimental unit. For the analysis of temperature and survival, treatment and survival (0 = yes, 1 = no) were included as fixed effects and batch as a random effect. For the analysis of IGF-1, treatment was included as fixed effect. For the figure the package ggplot was used in R. The level of statistical significance was set at *p* ≤ 0.05 and *p* < 0.10 was considered a tendency.

## 3. Results

During the experiment, 98 piglets out of 237 died or were removed from the nurse sow (41%) and thereby excluded from the dataset. In addition, 15 piglets were weaned before day 28 and these were also excluded. A total of 124 piglets were therefore included in the study on growth parameters until day 28, but all 237 IUGR piglets (WATER; *n* = 58, INJECT; *n* = 59, GLUC6; *n* = 60, GLUC12; *n* = 60) were included in the initial measurements.

### 3.1. Body Weight

**Figure 1:**
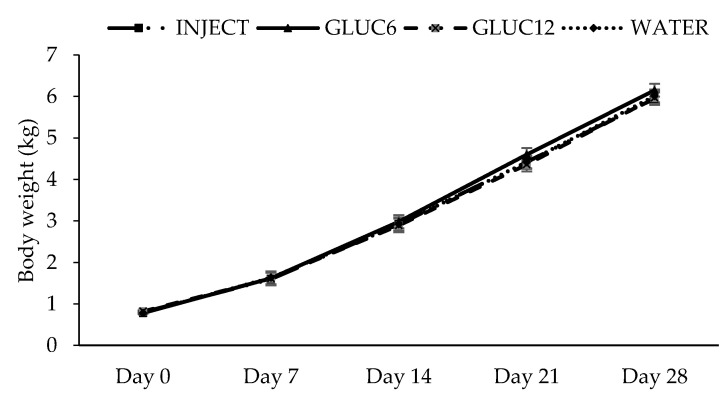
The body weight during lactation (until day 28) for the four treatments;WATER: 2 × 6 mL oral water (control) (*n* = 29)), INJECT: 2 × 6 mL glucose injected (*n* = 33), GLUC6: 2 × 6 mL oral glucose (*n* = 31), and GLUC12: 2 × 12 mL oral glucose (*n* = 31). Results are presented as lsmeans ± SEM.

Piglets in WATER, INJECT; GLUC6 and GLUC12 had similar mean birth weights of 0.78 ± 0.17 kg, 0.80 ± 0.16 kg, 0.78 ± 0.16 kg and 0.82 ± 0.16 kg at 0 h, respectively (*p* > 0.10). There was an effect of time (*p* < 0.001) with increasing body weights over days, but no effect of treatment (*p* = 0.281) or treatment × time interaction (*p* = 0.959; Figure 1).

### 3.2. Temperature, Blood Glucose Level and IGF-1

Whole-blood glucose levels were highest in the INJECT group at + 3 h compared to the other treatments WATER, GLUC6 and GLUC12 (*p* < 0.01; Table 1) respectively. There was no effect of treatment on the rectal temperature at 0 h or + 3 h (Table 1). The IGF-1 levels at day 7 were 37.0 ± 4.2 ng/mL, 29.6 ± 3.7 ng/mL, 35.6 ± 4.2 ng/mL, 33.3 ± 4.0 ng/mL (*n* = 9, 12, 9, 10) for WATER, INJECT, GLUC6 and GLUC12 respectively (*p* = 0.564).

### 3.3. Survival Rate

**Figure 2:**
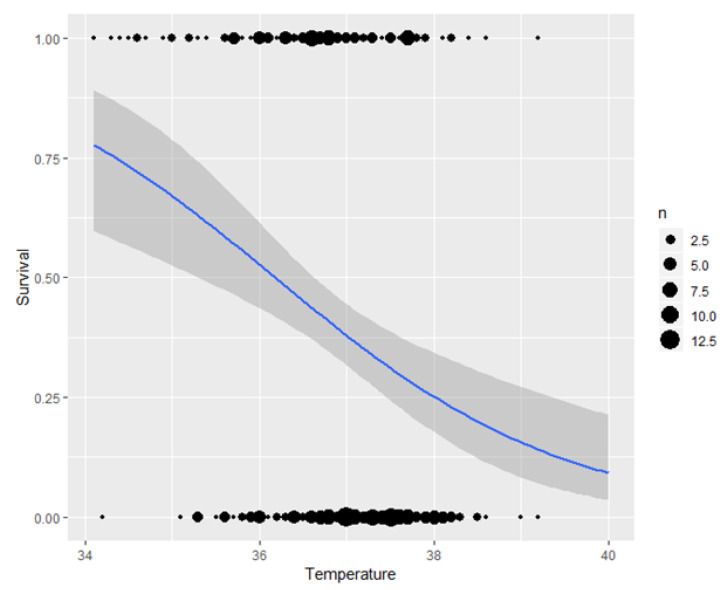
The rectal temperature (C°) at 0 h of piglets that survived (0) and died (1).

The odds ratio estimate for risk of dying or removal from the nurse sow during lactation was similar between groups (*p* = 0.19). Figure 2 shows the relationship between piglets dying or surviving and rectal temperature at 0 h. The average temperature for the piglets that died or survived were 36.6 ± 0.1 °C and 37.1 ± 0.1 °C respectively (*p* < 0.001). There was no difference in blood glucose between piglets that survived (3.2 ± 0.2 mmol/L) and piglets that died (2.7 ± 0.2; *p* = 0.134). There was no difference in blood glucose or rectal temperature between treatments at 0 h (*p* > 0.10).

**Figure 3:**
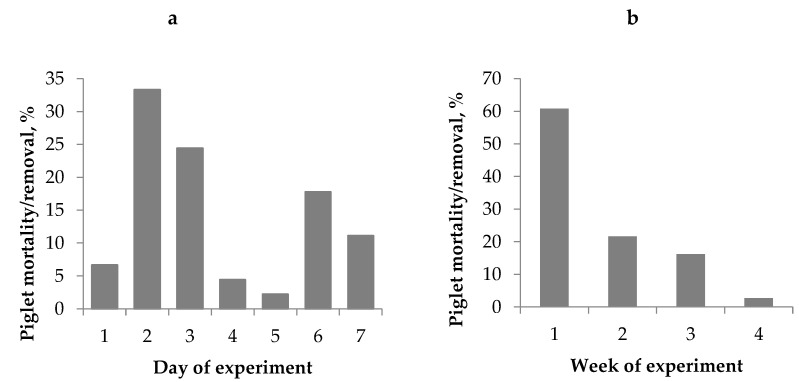
(**a**) Distribution of piglet deaths or being removed in the first week (*n* = 45); (**b**) Distribution of piglet deaths or removal over the experimental time period for all piglets (*n* = 74).

When a piglet was removed from the nurse sow it was recorded as being removed by the farm staff and the reason behind was registered. Some recordings were missing, therefore data is shown from 74 of the 98 piglets that were removed or died during the experiment. The majority of piglets that died or were removed occured within the first few days as can be seen in Figure 3a (*n* = 45). Piglet mortality/removal decreased after the first week (Figure 3b). Out of the 74 piglets, 24 were recorded as crushed, 26 as weak, 13 as having leg problems, 2 having diarrhea and 9 were recorded as other reasons.

## 4. Discussion

We hypothesized that giving glucose to IUGR piglets at litter equalization could increase survival rate and BW during lactation. Furthermore, that oral glucose would be equally effective as glucose injections. Results however, showed that the administration of glucose did not affect survival rate or BW gain during lactation when compared to piglets which received the control treatment (WATER). In order to mimic an on-farm situation, the piglets were given the treatments just before the farm staff would handle the piglets for the first time. This however, also meant that some piglets could be up to 20 h old if the sow had farrowed the previous day in the afternoon. In addition, the IUGR piglets were all placed together at a nurse sow in order to eliminate competition from larger littermates. This is a standard on-farm management practice in Denmark where up to 40% of sows can be used as nurse sows due to large litters [16].

Blood glucose level at + 3 h differed between groups where INJECT piglets had a higher blood glucose level compared to the other groups. Engelsmann et al. [14] also found an increase in blood glucose when giving an injection of glucose compared to a colostrum bolus. There was no difference in blood glucose level between WATER, GLUC6 or GLUC12 at + 3 h. The administration method determines the bioavailability of glucose and this differs when it is injected subcutaneously or given orally partly due to the insulin response [17]. It is possible that the bioavailability of blood glucose was also elevated in GLUC6 and GLUC12 piglets but this occurred earlier than + 3 h and had disappeared more rapidly (before + 3 h). However, it has been suggested that IUGR piglets have an impaired intestinal nutrient absorption surface, which may influence their ability to digest glucose [18] and this could also be an explanation.

At birth, piglets have innate energy reserves which mainly consist of glycogen and fat [19]. However, glycogen is the main substrate for oxidation after birth, because only a small amount of the fat can be utilized [19,20]. The glycogen reserves are small and quickly exhausted after birth especially in a cooler environment [19]. Declerck et al. [13] found that giving a commercial product consisting mostly of soya oil and coconut oil reduced mortality rate until day 21 in lactation in very low birth weight (<1.00 kg) and low birth weight (<1.20 kg) piglets. Similarly, Muns et al. [12] found that giving light birth weight piglets (<1.35 kg) a commercial product consisting mainly of glycerol and colostrum replacer decreased mortality rate the first 24 h after birth and mortality rate until day 21 in lactation, respectively. In contrast, Schmitt et al. [21] found that giving low birth weight piglets (<1.1 kg) coconut oil and a commercial product consisting mainly of fat did not affect survival rate or BW gain during lactation. The studies gave different doses of supplement (3 g in Declerck et al. [13]; 2 × 1 and 5 mL in Muns et al. [12]; 2 mL in Schmitt et al. [21]) equaling to different approximate total amount of energy (163 kJ in Declerck et al. [13]; 32 kJ and 83 kJ in Muns et al. [12]; 71 kJ in Schmitt et al. [21]). In comparison, the present study gave a higher dose of supplement to GLUC12 piglets (2 × 12 mL), however, this equals to a lower total amount of energy (19 kJ). According to Quesnel et al. [22], a piglet requires a minimum of 200 g of colostrum in order to significantly reduce the risk of mortality. This equals to 520 kJ (260 kJ/100 g; Theil el al. [23]) and suggests that supplementation of any product should only act as an energy booster. Furthermore, it suggests that the choice of supplement is secondary as long as it contains sufficient amount of energy. Nevertheless, it is uncertain, which amount of energy is sufficient to drive the piglet to the udder. The present study suggests that the amount of energy that piglets in the present study received was too low to have had an effect on survival rate and BW gain during lactation.

It is also important to consider when to give piglets supplement. Goodwin et al. [24] suggest that a glucose level below 2.8 mmol/L indicates hypoglycemia as this is the time where the pig starts to alter its behavior. In the present study, piglets were on average at 0 h above this value (2.9−3.4 mmol/L). However, from the raw data it can be seen that approximately 55% of all piglets at 0 h had a glucose level below this value (no difference between groups; *p* > 0.10), which indicates that piglets received the glucose supplement too late. In addition, the piglets that died did indeed have average blood glucose values of 2.7 mmol/L at 0 h indicating hypoglycemia compared to the piglets that survived (average blood glucose values of 3.2 mmol/L at 0 h) although no significant difference was found between the piglets. In Declerck et al. [13] and Muns et al. [12], piglets received a supplement at birth and within 4 h after birth, respectively. In the present study, piglets received glucose up to 20 h after birth which occurred after litter equalization. This was done, to simulate a commercial situation as on most commercial farms, managing IUGR piglets occurs sporadically and not immediately after birth given that the majority of sows farrow during the night.

Weaning weight was not different between treatments. In contrast, Engelsmann et al. [14] found that piglets injected with glucose had a higher weaning weight compared to piglets in the placebo group (5.39 vs. 4.57 kg; *p* < 0.05). However, in previous studies that have found differences [11,12,14] the treatment was either given at birth or up to 12 h later. It can be speculated that the WATER group was perhaps hydrated by the treatment. In addition, partly due to the sows being in free farrowing crates, the farm staff often gathered the piglets in the creep area to ensure that the piglets were kept warm. The farm’s strategy of providing warmth to all piglets was most likely beneficial for the IUGR piglets.

Piglets with a low rectal temperature (36.6 °C) at 0 h were more likely to die than IUGR piglets that had reached temperatures of 37.1 °C. This is in agreement with Baxter et al. [25] who also found that piglets dying postnatally had a much lower birth rectal temperature (dying = 36.5 °C vs. surviving = 37.7 °C). There was generally a low mortality rate amongst IUGR piglets (41%) compared to other studies [6]. Providing glucose to the small IUGR piglets is a management tool that requires a handling strategy and perhaps the increased attention is more important than the glucose in itself. In addition, some of the piglets that needed extra management care were most likely already dead within the first few hours and did therefore not even receive the experimental treatment.

## 5. Conclusions

In conclusion, the interventions at litter equalization in the current study (0 h) were too late in order to make a difference on growth and survival. Administering glucose orally and subcutaneously had a similar effect to the placebo group given a water bolus. Glucose injections increased blood glucose for a short term effect but with no lasting effects. Rectal temperature at 0 h was related to the survivability of the IUGR piglets.

## Figures and Tables

**Table 1:** The effect of treatment * on rectal temperature and whole blood glucose level. Results are presented as lsmeans ± pooled SEM.

Treatment	WATER	INJECT	GLUC6	GLUC12	SEM	*p*-Value
*n*	58	59	60	60		
Temperature at 0 h, °C	36.9	37.0	36.8	36.8	0.1	NS
Temperature at + 3 h, °C	37.4	37.3	37.2	37.3	0.1	NS
Glucose level at 0 h, mmol/L	3.4	2.9	2.9	2.9	0.6	NS
Glucose level at + 3 h, mmol/L	6.9 ^a^	12.6 ^b^	6.5 ^a^	7.3 ^a^	0.6	< 0.01

* WATER: 2 × 6 mL oral water (control), INJECT: 2 × 6 mL glucose injected, GLUC6: 2 × 6 mL oral glucose, GLUC12: 2 × 12 mL oral glucose. ^a,b^ Lsmeans in the same row with different superscript letters differ.

## References

[1] Milligan B.N., Fraser D., Kramer D.L. (2002). Within-litter birth weight variation in the domestic pig and its relation to pre-weaning survival, weight gain, and variation in weaning weights. Livest. Prod. Sci..

[2] Amdi C., Krogh U., Flummer C., Oksbjerg N., Hansen C.F., Theil P.K. (2013). Intrauterine growth restricted piglets defined by their head shape ingest insufficient amounts of colostrum1. J. Anim. Sci..

[3] Town S.C., Putman C.T., Turchinsky N.J., Dixon W.T., Foxcroft G. (2004). Number of conceptuses in utero affects porcine fetal muscle development. Reproduction.

[4] Chevaux E., Sacy A., le Treut Y., Martineau G. IntraUterine Growth Retardation (IUGR): Morphological and behavioural description. Proceedings of the 21st IPVS Congress.

[5] Roza S.J., Steegers E.A.P., Verburg B.O., Jaddoe V.W.V., Moll H.A., Hofman A., Verhulst F.C., Tiemeier H. (2008). What Is Spared by Fetal Brain-Sparing? Fetal Circulatory Redistribution and Behavioral Problems in the General Population. Am. J. Epidemiol..

[6] Hales J., Moustsen V.A., Nielsen M.B.F., Hansen C.F. (2013). Individual physical characteristics of neonatal piglets affect preweaning survival of piglets born in a noncrated system1. J. Anim. Sci..

[7] Lynegaard J.C., Hansen C.F., Kristensen A.R., Amdi C. (2019). Body composition and organ development of intra-uterine growth restricted pigs at weaning. Animals.

[8] Amdi C., Klarlund M., Hales J., Thymann T., Hansen C.F. (2016). Intra-uterine growth restricted piglets have similar gastric emptying rates but lower rectal temperatures and altered blood values than normal-weight piglets at birth. J. Anim. Sci..

[9] Holyoake P.K., Dial G.D., Trigg T., King V.L. (1995). Reducing pig mortality through supervision during the perinatal period. J. Anim. Sci..

[10] Dewey C.E., Gomes T., Richardson K. (2008). Field trial to determine the impact of providing additional care to litters on weaning weight of pigs. Can. J. Vet. Res. Rev. Can. Rech. Vet..

[11] Amdi C., Jensen L.L., Oksbjerg N., Hansen C.F. (2017). Supplementing newborn intrauterine growth restricted piglets with a bolus of porcine colostrum raises rectal temperatures one-degree Celsius1. J. Anim. Sci..

[12] Muns R., Nuntapaitoon M., Tummaruk P. (2017). Effect of oral supplementation with different energy boosters in newborn piglets on pre-weaning mortality, growth, and serological levels of IGF-I and IgG1. J. Anim. Sci..

[13] Declerck I., Dewulf J., Decaluwé R., Maes D. (2016). Effects of energy supplementation to neonatal (very) low birth weight piglets on mortality, weaning weight, daily weight gain and colostrum intake. Livest. Sci..

[14] Engelsmann M.N., Hansen C.F., Nielsen M.N., Kristensen A.R., Amdi C. (2019). Glucose Injections at Birth, Warmth and Placing at a Nurse Sow Improve the Growth of IUGR Piglets. Animals.

[15] Islam S., Piggott D.A., Moriggia A., Astemborski J., Mehta S.H., Thomas D.L., Kirk G.D. (2019). Reducing injection intensity is associated with decreased risk for invasive bacterial infection among high-frequency injection drug users. Harm Reduct. J..

[16] Bruun T., Amdi C., Vinther J., Schop M., Strathe A.B., Hansen C.F. (2016). Reproductive performance of “nurse sows” in Danish piggeries. Theriogenology.

[17] Elrick H., Stimmler L., Hlad C.J., Arai Y. (1964). Plasma Insulin Response to Oral and Intravenous Glucose Administration1. J. Clin. Endocrinol. Metab..

[18] D’Incà R., Che L., Thymann T., Sangild P., Luron I. (2010). Intrauterine growth restriction reduces intestinal structure and modifies the response to colostrum in preterm and term piglets. Livest. Sci..

[19] le Dividich J., Rooke J.A., Herpin P. (2005). Nutritional and immunological importance of colostrum for the new-born pig. J. Agric. Sci..

[20] Theil P., Nielsen M.O., Sørensen M., Lauridsen C., Bach K.E., Knudsen N.J., Kjeldsen H.D., Jensen B.B. (2012). Lactation, milk, and suckling. Nutritional Physiology of Pigs.

[21] Schmitt O., Baxter E., Lawlor P.G., Boyle L., O’Driscoll K. (2019). A Single Dose of Fat-Based Energy Supplement to Light Birth Weight Pigs Shortly After Birth Does Not Increase Their Survival and Growth. Animals.

[22] Quesnel H., Farmer C., Devillers N. (2012). Colostrum intake: Influence on piglet performance and factors of variation. Livest. Sci..

[23] Theil P.K., Lauridsen C., Quesnel H. (2014). Neonatal piglet survival: Impact of sow nutrition around parturition on fetal glycogen deposition and production and composition of colostrum and transient milk. Animals.

[24] Goodwin R.F.W. (1957). The relationship between the concentration of blood sugar and some vital body functions in the new-born pig. J. Physiol..

[25] Baxter E., Jarvis S., D’Eath R., Ross D., Robson S., Farish M., Nevison I., Lawrence A., Edwards S. (2008). Investigating the behavioural and physiological indicators of neonatal survival in pigs. Theriogenology.

